# An Ontology-Based Approach for Understanding Appendicectomy Processes and Associated Resources

**DOI:** 10.3390/healthcare13010010

**Published:** 2024-12-24

**Authors:** Nadeesha Pathiraja Rathnayaka Hitige, Ting Song, Steven J. Craig, Kimberley J. Davis, Xubing Hao, Licong Cui, Ping Yu

**Affiliations:** 1 Department of Information and Communication Technology, Faculty of Technology, Rajarata University of Sri Lanka, Mihintale 50300, Sri Lanka; ngtpr233@uowmail.edu.au; 2 Centre for Digital Transformation, School of Computing and Information Technology, University of Wollongong, Wollongong, NSW 2522, Australia; tsong@uow.edu.au; 3 Department of Surgery, Shoalhaven District Memorial Hospital, Nowra, NSW 2541, Australia; steven@drstevencraig.com.au; 4 Graduate School of Medicine, Faculty of Science Medicine and Health, University of Wollongong, Wollongong, NSW 2522, Australia; kimberley.davis@health.nsw.gov.au; 5 Research Operations, Illawarra Shoalhaven Local Health District, Warrawong, NSW 2502, Australia; 6 McWilliams School of Biomedical Informatics, The University of Texas Health Science Center at Houston, Houston, TX 77030, USA; xubing.hao@uth.tmc.edu (X.H.); licong.cui@uth.tmc.edu (L.C.)

**Keywords:** ontology, appendicectomy, surgical process, resources, process mining

## Abstract

Background: Traditional methods for analysing surgical processes often fall short in capturing the intricate interconnectedness between clinical procedures, their execution sequences, and associated resources such as hospital infrastructure, staff, and protocols. Aim: This study addresses this gap by developing an ontology for appendicectomy, a computational model that comprehensively represents appendicectomy processes and their resource dependencies to support informed decision making and optimise appendicectomy healthcare delivery. Methods: The ontology was developed using the NeON methodology, drawing knowledge from existing ontologies, scholarly literature, and de-identified patient data from local hospitals. Results: The resulting ontology comprises 108 classes, including 11 top-level classes and 96 subclasses organised across five hierarchical levels. The 11 top-level classes include “clinical procedure”, “appendicectomy-related organisational protocols”, “disease”, “start time”, “end time”, “duration”, “appendicectomy outcomes”, “hospital infrastructure”, “hospital staff”, “patient”, and “patient demographics”. Additionally, the ontology includes 77 object and data properties to define relationships and attributes. The ontology offers a semantic, computable framework for encoding appendicectomy-specific clinical procedures and their associated resources. Conclusion: By systematically representing this knowledge, this study establishes a foundation for enhancing clinical decision making, improving data integration, and ultimately advancing patient care. Future research can leverage this ontology to optimise healthcare workflows and outcomes in appendicectomy management.

## 1. Introduction

Surgical processes, while being a significant revenue source for hospitals, are also associated with considerable expenses. For example, the need for specialised surgical equipment and trained personnel can lead to high operational costs, while the procedures themselves can be major revenue drivers through insurance reimbursements and patient payments [1,2]. This dual nature requires the standardisation of surgical processes to balance cost efficiency [3,4]. Appendicectomy, a surgical removal of an inflamed appendix, was the most common emergency surgery in Australian public hospitals between 2014 and 2015, with 40,752 hospital admissions [5]. In New South Wales (NSW), Australia, 60% of these surgeries were publicly funded, indicating their substantial budgetary implications [6,7]. Consequently, it is imperative to optimise the appendicectomy processes for cost reduction, resource management, improved patient outcomes, and enhanced patient care.

Advanced process analysis techniques, such as process mining and business intelligence, are required to achieve the above objectives [8,9]. Process mining is a combination of data mining and business process management that analyses electronic health records (EHRs) using models that represent the process flow. This analysis is based on the sequence of events, their timing, and the assessment of resources used to infer the underlying diagnostic, treatment, and management processes [10]. Business intelligence, on the other hand, uncovers hidden patterns, correlations, health trends, patient behaviours, and other useful insights from extensive and diverse health datasets that encompass structured, semi-structured, and unstructured data [8,9]. However, the success of these healthcare process analysis techniques depends on a thorough understanding and accurate modelling of healthcare processes.

### 1.1. Challenges Associated with Healthcare Process Analysis

Successful healthcare process analysis and optimisation often require combined expertise in healthcare, hospital administration, and computation [11]. For example, surgical knowledge is required to explain the surgical procedural details to the project team. Hospital administrative staff contribute insights into resource management (e.g., scheduling operating rooms and staffing) and knowledge about legislative requirements in patient data handling, privacy, and organisational guidelines [4,12,13]. Meanwhile, information experts conduct data modelling to facilitate computational analysis. Therefore, an interdisciplinary approach is necessary for these projects [14,15]. To ensure effective communication among a multi-disciplinary project team, it is essential to develop a shared conceptual framework.

### 1.2. Modelling Approach to Healthcare Process Analysis

Two major healthcare modelling frameworks, openEHR and HL7, provide structured approaches to healthcare process modelling. OpenEHR emphasises the creation of reusable data structures by using a dual-model approach consisting of a Reference Model for technical consistency and Archetype Models for domain-specific content [16]. It is designed for capturing and storing structured, interoperable clinical data but lacks native support for semantic reasoning. OpenEHR employs models for clinical concepts, including surgical procedures, using archetypes and templates [17]. The archetype ACTION.procedure represents clinical activities performed for various purposes, including diagnostic and therapeutic interventions. The CLUSTER operative procedure records specific details of surgical interventions. It includes elements of Episode, Approach, Closure, Operative Diagnosis, and Outcomes. These archetypes are further organised into templates to support use-case-specific documentation. In contrast, HL7 focuses on syntactic and semantic clinical data exchange across systems. HL7’s Clinical Information Modelling Initiative (CIMI) includes models for procedures that emphasise semantic interoperability [18]. Within the CIMI, the “SurgicalProcedure” class focuses on attributes like surgical approach, access route, and implants, aligning with its goal of facilitating standardised data exchange rather than the granular, domain-driven modelling characteristic of openEHR.

### 1.3. Use of Ontology in Healthcare Process Mining

An ontology is a computer-readable, formal, explicit specification of shared knowledge within a particular domain [19]. Semantic ontology focuses on defining concepts, their attributes, and relationships within a domain, enabling semantic interoperability, reasoning, and knowledge representation [20]. Differing significantly from OpenEHR and HL7 CIMI in goals, modelling paradigms, and scope, semantic web ontology prioritises universal interoperability, knowledge integration, and automated reasoning using RDF/OWL, enabling rich semantics and cross-domain usability. While openEHR and HL7 CIMI and FHIR frameworks emphasise syntactic interoperability and system implementation, semantic web ontologies provide broader abstraction, nuanced granularity, and reasoning capabilities.

Ontologies have been successfully used to achieve semantic interoperability across multiple EHRs [21] and for clinical data representation in EHRs [22]. Examples include SNOMED CT and ICD, which deliver comprehensive vocabularies to consistently unify and represent clinical information across healthcare systems [23,24]. Ontologies are also used for modelling clinical pathway workflows [25,26,27].

In surgical process modelling, there remains a significant gap in understanding how to standardise and understand these processes effectively, particularly in appendectomies. For instance, Ye et al. proposed an ontology-based hierarchical semantic modelling approach to clinical pathway workflows, showing the potential of ontologies in structuring and standardising healthcare processes [25]. Another significant contribution is the Ontology for Surgical Process Models (OSPM), developed by Gibaud et al. [26]. This well-known surgical ontology provides a structured understanding of various surgical procedures, including laparoscopic adrenalectomies, cholecystectomies, and pancreatic resections [27]. However, the application of these ontological models to appendectomies remains underexplored. Therefore, there is a need to develop a novel ontology-based approach to understanding appendicectomy processes and associated resources.

Healthcare systems globally face mounting pressure from the surge in chronic diseases and rapid population growth [28,29]. Despite extensive research on healthcare resource management, a critical gap remains in understanding how to integrate and optimise these resources effectively to improve patient care whilst reducing costs. For instance, Olaronke et al. proposed an ontology-based remote patient monitoring framework for the Nigerian healthcare system, highlighting the need for semantic integration to improve interoperability and decision making in resource utilisation [28]. Similarly, Naseer et al. explored resource discovery in health grids, emphasising the importance of addressing resource heterogeneity [29]. They suggested that semantic ontology models could provide structured taxonomies for health grid resources, increasing access, interoperability, and resource sharing. These studies underscore the importance of semantic integration in healthcare resource management, particularly through collaborative efforts to develop standardised ontologies. Such frameworks play a pivotal role in structuring and standardising knowledge in healthcare, with significant implications for surgical data science and resource sharing.

Ontologies are essential tools for process mining, enabling the extraction of meaningful patterns from electronic health records (EHRs). They provide insights into surgical pathways, resource utilisation, and opportunities for process optimisation [25,26]. By integrating with clinical decision support systems, ontologies offer standardised representations of surgical workflows, supporting evidence-based decision making and promoting best practises [30]. Furthermore, they serve as educational tools, helping non-healthcare professionals like data analysts understand surgical processes and workflows [27]. Healthcare institutions can use ontologies for benchmarking surgical processes, identifying deviations, and driving quality improvement initiatives [26].

Despite the potential, no existing ontology specifically represents the appendicectomy processes and associated resources. Therefore, this research addresses this gap by developing an appendicectomy ontology to support interdisciplinary collaboration, enhance surgical process analysis, facilitate clinical decision making, and ultimately improve patient care.

## 2. Materials and Methods

### 2.1. Ethics Approval

The study protocol was reviewed by the Ethics Committee of the Illawarra Shoalhaven Local Health District (ISLHD), and the Committee classified this research as a Low and Negligible Risk project (Reference No.: ISLHD/LNR/2020-071) and can be executed with the acquisition of de-identified patient data.

### 2.2. Ontology Development Method

Various ontology development methodologies adhere to the principles of reusability and semantic stability [31]. These include but are not limited to Gruninger and Fox’s Methodology, the NeON Methodology, METHONTOLOGY, and the Uschold and King Methodology [32]. Gruninger and Fox’s methodology, METHONTOLOGY, and the Uschold and King Methodology are well established and designed for the development and evaluation of ontologies. Gruninger and Fox’s methodology, while effective in its design for enterprise engineering, does not cater to the specific needs of healthcare domains [33]. METHONTOLOGY, although robust, presents a challenge in its concept elicitation process due to a lack of formality [34,35]. For instance, the “Conceptualisation” stage in METHONTOLOGY involves the informal representation of domain terms in the form of concepts, instances, relationships, and properties [34,35]. The Uschold and King methodology, while comprehensive, is heavily scenario-dependent and does not prioritise collaboration [36,37]. In contrast, the NeON methodology excels in these areas [38,39]. It fosters collaborative ontology development, a critical requirement for formalising and interpreting surgical processes that necessitate collaboration across healthcare, administrative, and computational experts. Additionally, the NeON methodology promotes the reuse of both ontological and non-ontological resources and provides flexible pathways and activities for diverse scenarios. The NeON methodology has also been effectively utilised in various contexts, including the conceptualisation of a knowledge-driven approach for designing data analytics platforms [40] and understanding different time-related states observed in dynamic innovation ecosystems [41]. Therefore, given these considerations, the NeON methodology was selected for developing the target ontology in this research.

**Figure 1:**
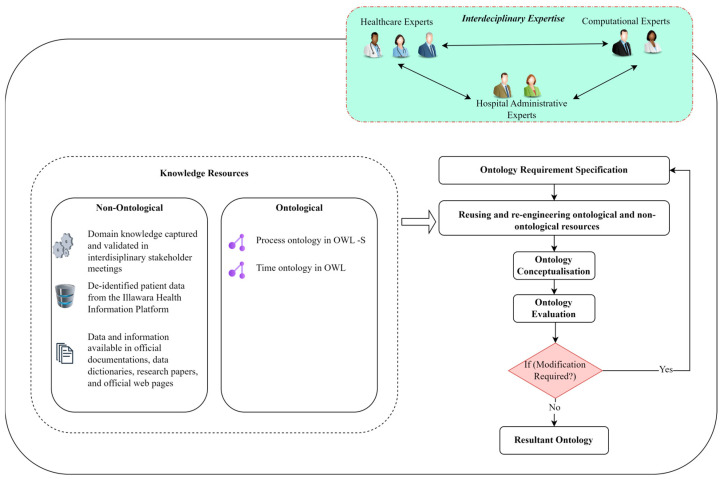
Proposed ontology development method (based on the NeON methodology) [38,42,43].

The NeON ontology development process includes four key iterative steps: ontology requirement specification, ontological and non-ontological resource reusing, ontology conceptualisation, and ontology evaluation and refinement (see Figure 1). Eight experts with specialised experience and knowledge in the relevant domains participated in the ontology development process. These experts included five knowledge engineering experts (P.Y., N.P.R.H., T.S., X.H., L.C.), a surgeon (S.J.C.), a hospital clinical researcher (K.J.D.), and an administrator.

Step 1: Ontology Requirement Specification

First, based on a review of the relevant literature and discussions within the research team, the basic requirements for the ontology were identified. These requirements are as follows: (1) its scope is to include knowledge of the appendicectomy processes and associated resources; (2) its intended users are knowledge engineers developing ontological resources related to surgical processes, and software engineers or interdisciplinary researchers conducting knowledge-driven process analysis, optimisation, and management of appendectomies; (3) its intended use cases are to support the understanding of appendicectomy processes and associated resources, to inform multidisciplinary researchers about the vast scope of data that need be considered in the appendicectomy process analysis, optimisation, and management projects; and (4) it is designed to answer the 11 competency questions prepared and validated by the domain experts and listed in Table 1.

Step 2: Ontological and Non-Ontological Resource Reuse

To extract knowledge related to the appendicectomy pathway, four key resources were utilised: existing ontologies; clinical models; hospital guidelines, policies, and procedures; and de-identified appendicectomy patient data from the Illawarra health information platform. Eash resource contributed unique insights, ensuring a comprehensive and clinically relevant representation of the appendicectomy process.

First, five existing ontologies were reviewed and reused to ensure compatibility, comprehensiveness, and semantic accuracy in modelling the appendicectomy pathway: (1) Process ontology in OWL-S [44], which provides a computer-interpretable framework for processes, applicable for modelling the appendicectomy pathway. (2) The time ontology in OWL [45]. This is built upon temporal logic and enables the representation of time-bound aspects of the clinical pathway, such as procedure scheduling and duration [46,47]. (3) SNOMED CT, which offers comprehensive clinical terminology essential for describing medical conditions, procedures, and outcomes related to appendicectomy [48,49]. (4) The Foundational Model of Anatomy (FMA), which provides detailed anatomical information crucial for mapping the surgical process [48,50].

Second, clinical models from OpenEHR and the HL7 Clinical Information Modelling Initiative (CIMI) [51,52,53,54] were critically analysed for their structured representation of surgical procedures, including preoperative preparation, surgical steps, and postoperative care. The OpenEHR operative procedure archetype provides templates that document the entire surgical timeline [17], including the scheduled date/time for precise coordination, the end date/time for calculating total surgery duration, and the action time attribute for tracking critical events such as incision and procedure completion. This detailed representation enables accurate monitoring of surgical processes, supporting postoperative care, auditing, and quality improvement.

Third, hospital guidelines, policies, and procedures were reviewed to derive organisational knowledge related to appendicectomy, associated EHRs, and data variables. These documents include the NSW Operating Theatre Efficiency Guideline [4], NSW Emergency Surgery Guidelines and Principles for Improvement [55], Privacy Issues and the Reporting of Small Numbers [56], and the Royal Australasian College of Surgeons’ Surgical Audit Guide 2021 [57]. Reference was also made to health data dictionaries held by the Centre for Health Research Illawarra Shoalhaven population and authoritative websites. These resources provided insight into operational procedures, data variables, and ethical considerations in patient data handling. Health data dictionaries and authoritative websites on emergency surgeries and appendectomies were also references to ensure alignment with current practice and regulatory requirement [58,59,60].

Fourth, de-identified electronic medical record (EMR) data for 3943 patients who underwent appendicectomy between January 2014 and July 2020 at the two designated hospitals were sourced from the Illawarra Health Information Platform. This dataset included admitted patient data (527 variables), emergency department (ED) data (37 variables), medical imaging data (16 variables), pathology data (37 variables), and theatre data (42 variables). These de-identified data served as the ‘ground truth’ for validating the ontology, ensuring its accuracy, clinical relevance, and practical utility.

Step 3: Ontology Conceptualisation

The ontology class creation process was a collaborative effort of the multidisciplinary research team. The process started with a comprehensive analysis of existing ontological and non-ontological resources to identify potential classes, properties, and data values. Relevant ontologies, such as SNOMED CT and FMA, were referenced for concepts like anatomical structures (e.g., the appendix) and surgical procedures (e.g., laparoscopic appendicectomy) [48,49,50]. Concepts not represented in the literature, such as appendicectomy-specific care protocols and patient outcomes, were identified for new class creation.

Classes and their properties were developed iteratively, with the team meeting regularly to refine definitions and relationships. This included categorising properties into object properties (to establish entity relationships) and data properties (to link entities with values). The refined ontology was formalised using Web Ontology Language 2 (OWL 2) and implemented in Protégé 5.5.0, an open-source ontology editor [44,61]. The finalised ontology, stored in XML/RDF format, was designed for compatibility with various systems and applications.

Step 4: Ontology Evaluation and Refinement

The quality of the ontology was evaluated through three comprehensive steps. First, the domain experts assessed the ontology’s accuracy, clarity, completeness, and conciseness using a semi-structured interview guide (Appendix A). This step involved iterative refinement by integrating feedback from the multidisciplinary team. Second, a combination of automated and manual methods was used to ensure technical robustness and functional applicability. The OntOlogy Pitfall Scanner was used by the knowledge engineers to detect errors such as undeclared inverse relationships in ontologies [62]. Meanwhile, 11 competency questions were transformed into SPARQL queries to test whether the ontology met the specified requirements by the digital health experts [63]. These semantic queries evaluated the ontology’s ability to represent key clinical scenarios and processes effectively.

Finally, the ontology was validated using real-world EHR data and process mining techniques. Patient data were mapped onto the ontology to instantiate the model, enabling practical validation of its representational capacity. Logical consistency checks and ontology-driven queries were conducted to ensure alignment with clinical workflows and data accuracy. The results were benchmarked against traditional EHR query methods to assess improvements in efficiency and insights. Use case applicability was further tested by a process mining project, with outcomes evaluated by a clinical research manager and two surgeons for clinical relevance and acceptability [64].

## 3. Results

### 3.1. The Resultant Ontology

**Figure 2:**
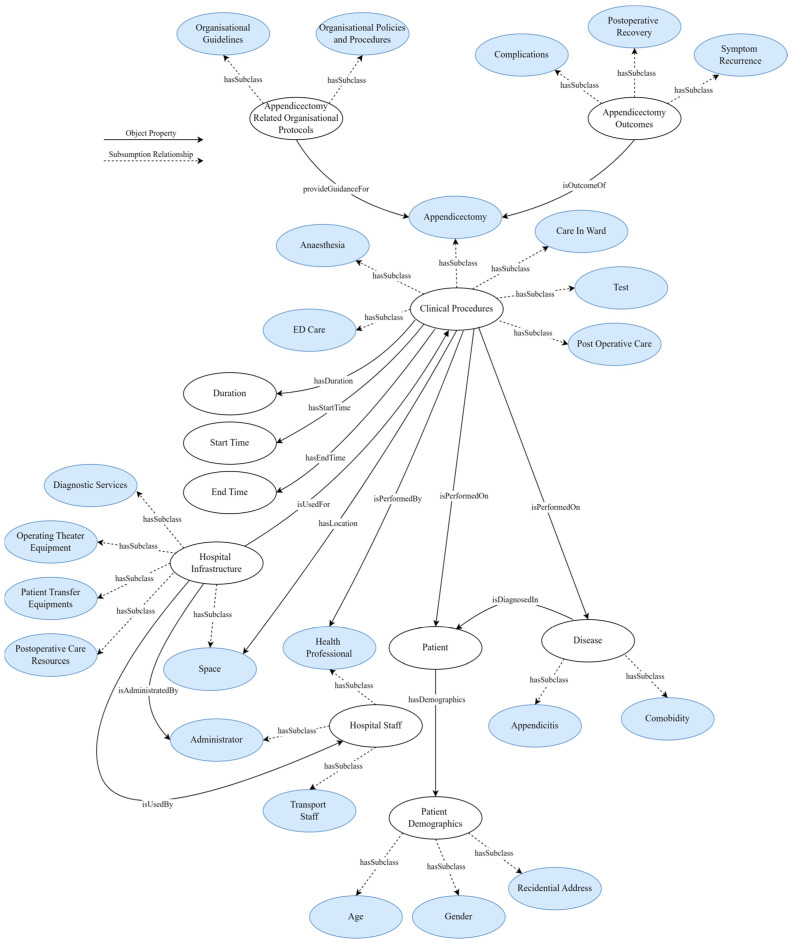
A simplified ontology graph of appendicectomy process and resource ontology with major classes and their relationships.

**Figure 3:**
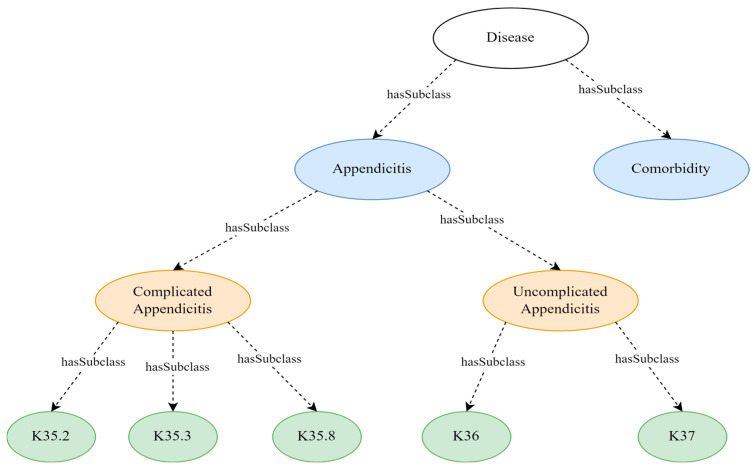
Simplified disease class with major subclasses and their relationships.

**Figure 4:**
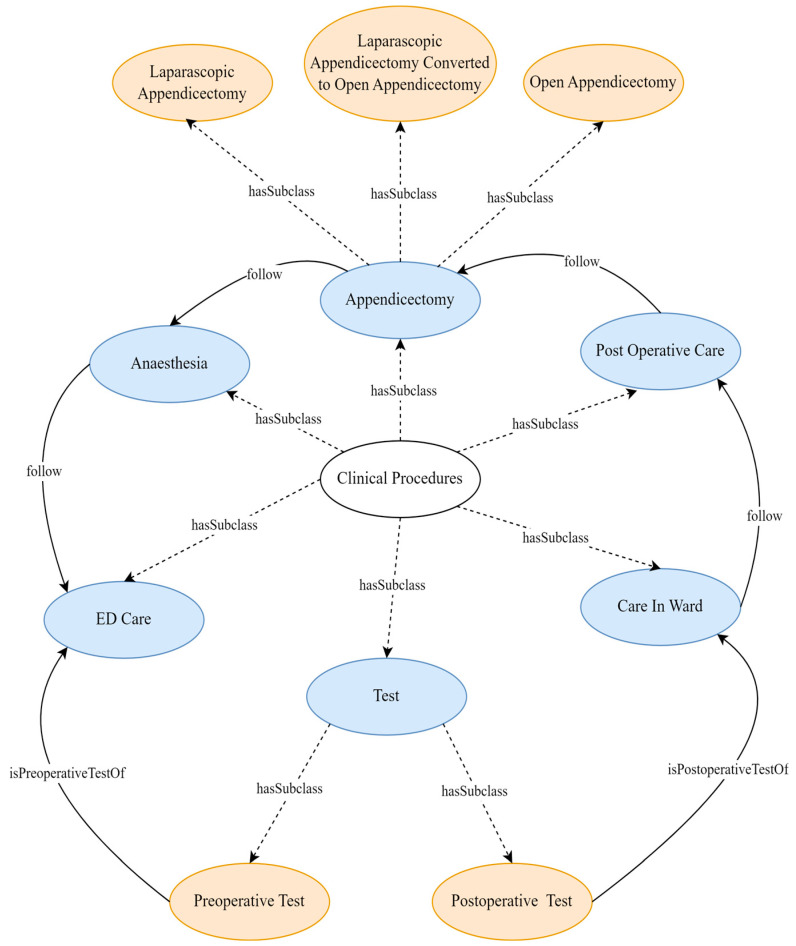
Simplified clinical procedure class with major subclasses and their relationships.

The resultant ontology has 108 classes. There are 11 classes at the top level: “clinical procedure”, “appendicectomy-related organisational protocols”, “disease”, “start time”, “end time”, “duration”, “appendicectomy outcomes”, “hospital infrastructure”, “hospital staff”, “patient”, and “patient demographics” (see Table 2 and Figure 2, Figure 3 and Figure 4). The maximum depth is four level subclasses. The top-level class “disease” contains two subclasses “appendicitis” and “comorbidity”. Moreover, 77 object and data properties were created to establish relationships between the classes.

### 3.2. The Core of the Resultant Ontology

The ***“disease”*** ontology class encapsulates appendicitis and comorbidities diagnosed in appendicectomy patients (see Figure 3). The subclass “appendicitis” consists of “complicated appendicitis” and “uncomplicated appendicitis”. Subclasses of these two types of appendicitis are represented by the International Classification of Diseases, Tenth Revision, Australian Modification (ICD—10 AM) codes. The “comorbidity” class represents the other diseases diagnosed in appendicectomy patients other than appendicitis.

The ***“clinical procedure”*** ontology class, which represents the sequence of activities performed during appendectomies, is divided into six direct subclasses: “ED care”, “anaesthesia”, “appendicectomy”, “care in ward”, “postoperative care”, and “test” (see Figure 4). Each of these clinical procedures has a “start time”, “end time”, and “duration” (see Figure 2). Furthermore, to perform these clinical procedures, it requires “hospital infrastructure” and is performed by a “health professional”. The “ED care” subclass encompasses the steps taken to prepare a patient for surgery, including emergency department procedures to surgery booking. The “test” subclass represents the preoperative and intraoperative tests that can be performed during the “ED care” or “care in ward” procedures. The “appendicectomy” subclass refers to the actions taken during the surgery itself, such as the surgical procedures to remove the appendix, and it has three subclasses “laparoscopic appendicectomy”, “laparoscopic appendicectomy converted to open appendicectomy”, and “open appendicectomy”. The “postoperative care” subclass involves the care provided immediately after the surgery, including post-anaesthetic care. Finally, the “care in ward” subclass involves the care provided to the patient in the ward until they are ready to be discharged from the hospital.

The ***“appendicectomy-related organisational protocols”*** ontology class encapsulates the guidelines, policies, and procedures established by healthcare organisations for the effective management of appendectomies. It has two direct subclasses, “organisational guidelines” and “organisational policies and procedures” (see Figure 2). Each of these classes consists of the relevant individuals, which are mainly organisational documents that guide support hospitals, local health districts, and specialty health networks in planning their appendectomies based on a predictable long-term workload. This includes emergency surgical service plans, capacity assurance techniques, variation minimisation approaches, and improvement monitoring mechanisms. Incorporating these protocols is essential for guiding the appendectomies towards the optimisation and management of the surgical processes. The ***“appendicectomy outcomes”*** ontology class represents the outcomes of appendicectomy (see Figure 4). It has three direct subclasses: “complications”, “postoperative recovery”, and “symptom recurrence”.

**Figure 5:**
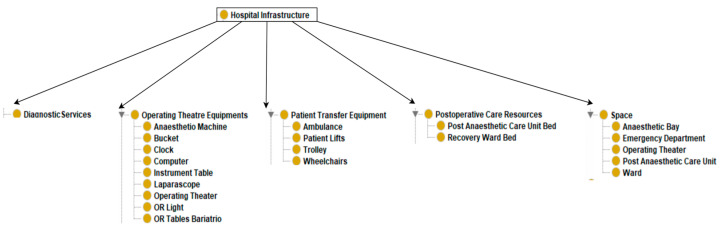
Hospital infrastructure class.

**Figure 6:**
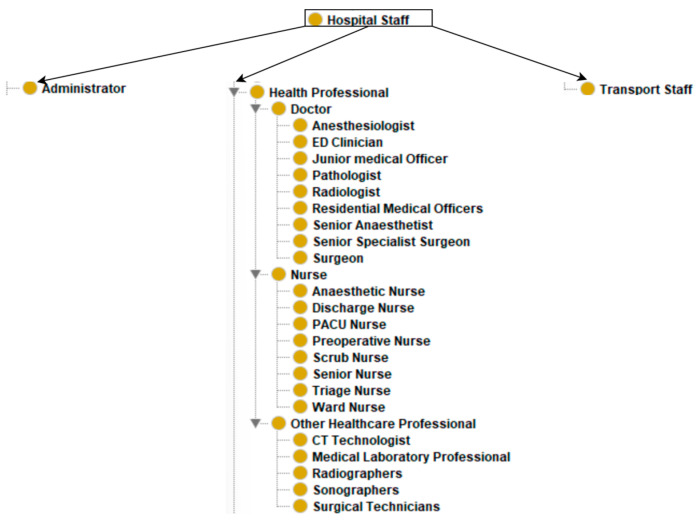
Hospital staff class.

The ontology class ***“hospital infrastructure”*** is a critical component in the appendicectomy pathway (see Figure 5). It encompasses a broad spectrum of hospital infrastructure, essential for the seamless execution of an appendicectomy. This infrastructure class encapsulates diagnostic services, operating theatre equipment, patient transfer system equipment, and resources dedicated to postoperative care and space. The ontology class ***“hospital staff”***, which represents the human resources involved in facilitating appendectomies while utilising the hospital infrastructure, is divided into three direct subclasses, “administrator”, “health professional”, and “transport staff” (see Figure 6). Administrators are responsible for administrating the hospital staff and hospital infrastructure, while healthcare professionals perform the clinical activities. These comprehensive classes of resources ensure that every aspect of the appendicectomy processes, from preoperative preparations to postoperative care, is adequately catered for, thereby facilitating a smooth and efficient surgical pathway.

### 3.3. Results of Evaluating the Ontology

The ontology was evaluated for consistency, clarity, completeness, conciseness, and accuracy. The reasoner Pellet’s evaluation results confirmed the logical consistency of the ontology. Regarding clarity, the domain experts identified labels or ambiguous definitions that were difficult to understand and replaced them with the recommended new terms and definitions. Furthermore, domain experts confirmed that ontology satisfied the first two requirements in the requirement specification section. The alignment between the anticipated data and the data inferred from the retrieval results, as per the SWRL rule, substantiates the third requirement, demonstrating the utility of the ontology. Regarding the fourth requirement, the ontology accurately addressed the competency questions, as evidenced by the SPARQL results. Furthermore, the usefulness of the ontology was validated by its implementation in a process mining project to uncover appendicectomy pathways in two public hospitals at ISLHD [64].

## 4. Discussion

This research contributes to integrating the domain-specific appendicectomy process details, including hospital infrastructure, staff roles, organisational policies, resources, protocols, and workflows, into a semantic framework, enabling knowledge integration and inferencing capabilities absent in openEHR and HL7’s CIMI [16,17]. The ontology’s structured representation of sequential surgical steps facilitates extracting meaningful insights from complex data. By addressing the recognised challenge of creating standard ontologies, this work supports data-driven approaches to improving surgical outcomes. It also emphasises an interdisciplinary approach, combining clinical, administrative, and computational expertise to optimise workflows and decision making [14,15]. Therefore, this research is in line with advancements in surgical data science [26].

Our ontology-based approach provides several distinct advantages over other methods, such as association rules, Bayesian networks, and classification trees, particularly for surgical process modelling [65,66,67]. Ontologies enable semantic interoperability, enabling data integration and standardisation across disparate systems and datasets, a critical need in healthcare. Unlike other methods, ontologies capture domain knowledge, including complex relationships, constraints, and workflows, ensuring high-fidelity modelling [68,69,70]. They also allow for flexible adaptation, allowing it to incorporate new concepts and adjust to evolving practises. This ensures the model remains relevant over time. Moreover, their reasoning capabilities enable them to derive new insights beyond pattern recognition, a limitation of methods like association rules or classification trees. Another key strength of ontologies lies in their grounding in international standards, promoting reusability, collaboration, and data sharing across institutions [71,72]. These characteristics make ontologies robust, adaptable, and uniquely suited to addressing the complexities of modelling surgical processes in diverse healthcare contexts.

The existing surgical process models, such as the Ontology for Surgical Process Models and the Surgical GPS ontology, primarily focus on procedural steps or specific guidance (e.g., scoliosis procedures) [73]. Conversely, our ontology provides a structured framework for integrating hospital infrastructure, staff roles, organisational policies, resources, protocols, and workflows, which facilitates the implementation of standardised protocols and best practises [74]. It also aligns closely with established guidelines and research, such as the SAGES Guideline for the Diagnosis and Treatment of Appendicitis [75]. Although tailored to appendicectomy, its foundational structure and methodology can be adapted to other surgical contexts by incorporating procedure-specific concepts and relationships. Future work could leverage its core design principles to address other surgical procedures.

Compared to studies on surgical task automation or robotics-related ontologies, which often lack a domain-specific focus, our ontology specifically models appendicectomy processes. Therefore, our ontology helps us understand real-world appendicectomy surgical processes and associated resources. It has been successfully employed to uncover processes of appendicectomy within two local public hospitals, generating novel insights into clinical workflows and resource allocation [64]. To address the evolving surgical procedures, the ontology requires ongoing updates to incorporate new concepts and remove obsolete ones for its continued alignment with current practises. Additionally, feedback from domain experts and iterative validation against real-world data are crucial to maintaining its accuracy, relevance, and confidence for application in dynamic clinical environments.

This study has four limitations. First, the ontology is tailored explicitly to appendicectomy processes and associated resources with the Australian public health system, limiting its direct applicability to other surgical procedures without further adaptation. Second, geographical and local variations influence the ontology’s development, including differences in clinical practises and data captured by various hospital information systems [64]. Therefore, caution should be taken when applying the ontology beyond its original context. Third, the ontology relies on domain knowledge extracted from the literature and local EHRs. Its accuracy and completeness depend on these data sources’ quality, availability, and comprehensiveness. As medical practises and associated resources evolve, regular updates will be essential to maintain the ontology’s relevance, necessitating effective update management. Fourth, the ontology is a conceptual representation of the appendicectomy surgical process, not a universal solution. To maximise its utility, a multidisciplinary approach involving healthcare professionals and administrators is recommended. This ensures that the ontology’s variables and data remain comprehensive, relevant, and practical for addressing challenges in surgical data science projects.

## 5. Conclusions

This research developed a novel ontology to represent appendicectomy processes and associated resources, addressing the critical gap in cross-platform knowledge integration and analytics in health informatics. By incorporating appendicectomy-specific details into a semantic framework, this study offers a robust tool for understanding and optimising surgical workflows. Its fine-grained structure not only facilitates a detailed analysis of appendicectomy procedures but also provides a foundation for expansion to related surgical contexts. The ontology’s flexible structure ensures adaptability to evolving practises, underscoring its relevance in dynamic clinical settings.

This research advances the conceptualisation and computerisation of surgical ontologies, paving the way for enhanced decision support, resource planning, and patient care. By addressing the inherent complexity of surgical processes, it highlights the transformative potential of ontologies in optimising operations and improving outcomes. This research’s unique focus on appendicectomy demonstrates the practical applicability of semantic framework-ontology, offering actionable insights and establishing a pathway for future research to revolutionise healthcare delivery.

## Figures and Tables

**Table 1:** Competency questions for the appendicectomy ontology to answer.

No. of CQ	Competency Questions (CQ)
1.	What are the clinical procedures associated with appendicectomy?
2.	What organisational protocols (guidelines, policies, and procedures) are related to appendicectomy?
3.	What are the appendicectomy outcomes?
4.	What are the common types of appendicectomy?
5.	What are the common types of appendicitis diagnosed in appendicectomy patients?
6.	What are the comorbidities existing in appendicectomy patients?
7.	What specific pieces of hospital infrastructure are required for appendicectomy?
8.	What hospital staff are required for appendicectomy?
9.	What are the roles of different health professionals in the appendicectomy pathway (who performs which activity)?
10.	What time parameters are used to describe how long an appendicectomy clinical procedure takes?
11.	What are the usual steps clinicians follow in executing an appendicectomy on a patient?

**Table 2:** Hierarchical distribution of classes with names of first-level classes and the number of classes at all levels.

Title 1	Number of Classes at Different Levels					Number in Total (with %)
	1st	2nd	3rd	4th	5th	
Appendicectomy-Related Organisational Protocols	1	2	0	0	0	3 (2.77%)
Appendicectomy Outcomes	1	3	0	0	0	4 (3.70%)
Clinical Procedure	1	6	5	5	11	28 (25.92%)
Disease	1	2	2	5	0	10 (9.25%)
Hospital Infrastructure	1	5	20	0	0	26 (24.07%)
Hospital Staff	1	3	3	22	0	29 (26.85%)
Patient	1	0	0	0	0	1 (0.92%)
Patient Demographics	1	3	0	0	0	4 (3.70%)
Start Time	1	0	0	0	0	1 (0.92%)
End Time	1	0	0	0	0	1 (0.92%)
Duration	1	0	0	0	0	1 (0.92%)

## Data Availability

The data analysed in this study are not publicly available by the conditions set by the Human Research Ethics Committee for this project, which prioritise participant privacy and confidentiality.

## Appendix A. Semi-Structured Interview Guide for Evaluating the Ontology

*Scope of the Ontology*

1. How well does the ontology capture the knowledge of the appendicectomy surgical process and associated resources?
2. Can you provide specific examples of how the ontology represents the appendicectomy surgical process and associated resources?
3. In what ways does the ontology accurately reflect the reality of appendicectomy surgical processes and associated resources?
4. Are there any aspects of the appendicectomy surgical process and associated resources that you feel are not adequately represented in the ontology?
5. How could the scope of the ontology be improved to better represent the appendicectomy surgical process and associated resources?

*Intended Users*

6. As a knowledge engineer/software engineer/interdisciplinary researcher, how useful do you find this ontology in developing ontological resources related to surgical processes or conducting knowledge-driven process analysis, optimisation and management of appendicectomy surgeries?
7. What features of the ontology do you find most useful in your work?
8. Are there any features or aspects of the ontology that you find challenging or difficult to use?
9. How does this ontology compare to other resources you have used in your work related to surgical processes?
10. What improvements would you suggest making the ontology more user-friendly or useful for its intended users?

*Use Cases*

11. Can you provide examples of how this ontology has supported your understanding of the appendicectomy surgical process and associated resources?
12. How has the ontology informed you about the scope of data that needs to be considered in appendicectomy process analysis, optimisation and management projects?
13. Can you describe a scenario where the ontology was particularly useful or effective in supporting your work?
14. Are there any use cases where the ontology did not meet your needs or expectations? If so, can you describe these situations?
15. What additional use cases do you think the ontology could support in the future?

*Competency Questions*

1. Can you provide examples of how the ontology answers the eleven competency questions?
2. How does the ontology meet the content-specific requirements represented in the form of natural language questions (the competency questions)?
